# Chronic traumatic encephalopathy neuropathologic change is associated with highest stage limbic-predominant age-related TDP-43 encephalopathy

**DOI:** 10.1093/jnen/nlag040

**Published:** 2026-04-27

**Authors:** Hailong Song, Kamar E. Ameen-Ali, Claire Kennedy-Dietrich, Jean-Pierre Dolle, Edward B. Lee, Douglas H. Smith, William Stewart, the CONNECT-TBI Investigators

**Affiliations:** 1 Department of Neurosurgery, Center for Brain Injury and Repair, University of Pennsylvania, Philadelphia, PA, United States; 2 School of Health and Life Sciences, Teesside University, Middlesbrough, United Kingdom; 3 School of Cancer Sciences, University of Glasgow, Glasgow, United Kingdom; 4 Translational Neuropathology Research Laboratory, University of Pennsylvania, Philadelphia, PA, United States; 5 Department of Neuropathology, Queen Elizabeth University Hospital, Glasgow, United Kingdom

**Keywords:** chronic traumatic encephalopathy neuropathologic change, limbic-predominant age-related TDP-43 encephalopathy, repetitive head impact, TDP-43, neuropathology, traumatic brain injury

## Abstract

Traumatic brain injury (TBI) is recognized as a major risk factor for neurodegenerative disease (NDD). Autopsy studies frequently describe chronic traumatic encephalopathy neuropathologic change (CTE-NC) in individuals with histories of repetitive head impact (RHI) exposure, often with accompanying comorbid neurodegenerative proteinopathies. Of these, deposition of abnormally phosphorylated TDP-43 (pTDP-43) has been reported but the prevalence and distribution of pTDP-43 in CTE-NC and its distinction from that encountered in wider NDD are uncertain. Here, patients with a history of RHI and documented NDD (*n* = 30), and age-matched controls with no known TBI or RHI exposure, either with (*n* = 24) or without (*n* = 18) NDD, were identified within the CONNECT-TBI archive. Standardized brain tissue sections stained for pTDP-43 were assessed. pTDP-43 pathology prevalence was similar among RHI patients (40%) and controls with NDD (33%). pTDP-43 was typically localized (limbic-predominant age-related TDP-43 encephalopathy neuropathologic change [LATE-NC] stage 1 to 2) in amygdala and hippocampus in controls with NDD and following RHI exposure without CTE-NC. In contrast, this pathology was often widespread and of high stage (LATE-NC stage 3; *P* = .0045) in patients with CTE-NC. Thus, CTE-NC may be associated with more widespread pTDP-43 pathology than encountered in aging or those with NDD and no history of TBI/RHI.

## Introduction

Traumatic brain injury (TBI) and exposure to repetitive head impacts (RHI) are recognized as major, potentially modifiable risk factors for a range of neurodegenerative diseases (NDDs), including Alzheimer disease (AD) and chronic traumatic encephalopathy (CTE).^1–4^ Among the complex neuropathologies described in TBI-related neurodegeneration (TReND), abnormal phosphorylation and localization of 43kDa transactive response DNA binding protein (TDP-43) have been documented in limited case series.^5–10^ Nevertheless, despite being included in consensus criteria as a supportive pathology for the diagnosis of CTE neuropathologic change (CTE-NC),^11^ the prevalence and distribution of TDP-43 pathology in those with histories of TBI/RHI and CTE-NC, and its distinction from that seen in association with aging and wider NDD without history of TBI/RHI, have not been fully assessed.

Since its first recognition as a major disease associated protein in association with frontotemporal dementia and amyotrophic lateral sclerosis (ALS),^12,13^ TDP-43 pathology has been increasingly recognized as a distinct neurodegenerative proteinopathy described as limbic-predominant age-associated TDP-43 encephalopathy (LATE).^14–16^ In isolation, LATE neuropathologic change (LATE-NC) is reported to be associated with an amnestic syndrome presenting at relatively older age and with slower symptom progression when compared to AD.^17,18^ However, LATE-NC is more frequently encountered as a co-morbid pathology in the context of mixed NDD pathologies,^19^ with LATE-NC present in up to 70% of those with otherwise typical AD pathology.^20,21^ In this setting, data to date suggest that patients with comorbid LATE-NC may show a more aggressive clinical course^20,21^ and a distinct symptom profile^22,23^ as compared to patients with isolated AD neuropathological change.

In the context of TBI, limited studies report abnormal deposition of phosphorylated TDP-43 (pTDP-43). First documented in context of dementia pugilistica in individuals with typically long careers in professional boxing,^24–26^ more recent studies have explored the association between pTDP-43 proteinopathy and TBI/RHI among those with non-boxing exposures.^8,10,27–29^ For example, a study by Saltiel et al reported 24.0% of cases with history of RHI showed pTDP-43 inclusions, whereas Nicks et al examined brains with CTE-NC at autopsy and found that 43.3% had pTDP-43 deposition in association with hippocampal sclerosis. Indeed, where there has been documented exposure to RHI, pTDP-43 pathology appears, at least in part, dependent on a finding of coexistent CTE-NC, ie present in cases where CTE-NC is present^5^ and absent where no CTE-NC is found.^30^ In contrast, following survival from a single moderate or severe TBI (sTBI), no clear evidence of pTDP-43 pathology has been described.^31^ However, cytoplasmic translocation of phosphorylation independent TDP-43 has been documented;^31,32^ perhaps refelcting a physiological response to injury.^33^

Thus, despite appearing as a supportive pathology in consensus definitions of CTE-NC,^11^ the prevalence and distribution of pTDP-43 as a component pathology contributing to TReND and its distinction from the pathologies of NDD currently remain uncertain. To address this, we examined unique case materials from the COllaborative Neuropathology NEtwork Characterizing ouTcomes of TBI (CONNECT-TBI) Archive,^34^ to test our hypotheses that (1) exposure to RHI results in increased risk of pTDP-43 pathology and (2) where present, pTDP-43 pathology in the context of RHI exposure will be more widely distributed than that seen in association with aging and NDD.

## Methods

### Cohort identification and demographics

Autopsies were conducted with informed consent from next of kin according to local laws and regulations. All cases and controls were identified within the multi-center CONNECT-TBI^34–36^ archive holdings as consecutive research brain donations fulfilling the requirements for each study group. All clinical histories were collected via a self- or proxy-report assessment. Inclusion criteria included patients with history of exposure to RHI (contact sports participation, military service, or multiple concussions) with or without history of NDD (and/or neurodegenerative pathology), patients with history of NDD but no known history of exposure to TBI/RHI, and patients with no known history of TBI/RHI or NDD. In this study, 3 groups were identified as: individuals with NDD and history of exposure to RHI (RHI + NDD) (*n* = 30) either with (*n* = 16) or without (*n* = 14) CTE-NC, as defined by consensus criteria for the neuropathological evaluation of CTE-NC with staged severity of low or high CTE-NC;^11^ age matched controls with NDD, but no documented RHI exposure (no RHI + NDD, *n* = 24); or age matched controls with no documented NDD or RHI exposure (no RHI or NDD, *n* = 18) (Table 1). In contrast to the control groups, nearly all brain donors from the RHI + NDD group (29/30, 96.7%) were male. Clinical, demographic, and neuropathological data for all cases and controls are presented in Table 2 and Table S1.

All procedures performed in this study involving human participants were in accordance with the ethical standards of the institutional and/or national research committee and with the 1964 Helsinki Declaration and its later amendments or comparable ethical standards. For this type of study, formal consent is not required. Tissue samples were obtained from the Glasgow TBI Archive, University of Pittsburgh, Icahn School of Medicine at Mount Sinai, Sick Kids Toronto, University of Washington, and University of Pennsylvania Center for Neurodegenerative Disease Research as part of the CONNECT-TBI. Tissue was acquired at routine diagnostic autopsy, and approval for its use was granted by the respective institutional review boards.

### Immunohistochemistry

Standard histological staining protocols were followed, as previously described.^34,37^ In brief, 8 *μ*m thick tissue sections were prepared from formalin-fixed paraffin processed tissue blocks selected to include standardized anatomical regions for assessment of LATE-NC,^16^ ie the middle frontal gyrus, the amygdala and the hippocampus at the level of the lateral geniculate nucleus, together with sections of the medulla. Sections were deparaffinized then rehydrated, prior to immersion in 0.3% H_2_O_2_ in methanol (15minutes) to quench endogenous peroxidases. Thereafter, sections were blocked and incubated with anti-pTDP-43 antibody (1D3; 1:500, Millipore) overnight at 4° C prior to incubation with a biotin-conjugated secondary antibody at room temperature, then visualization with the avidin–biotin complex detection method (VECTASTAIN ABC kit; Vector Laboratories, Burlingame, CA) with ImmPACT diaminobenzidine peroxidase substrate (Vector Laboratories) as the chromogen. Sections were then counterstained with Hematoxylin, digitally scanned with a 40x objective on an Aperio AT2 slide scanner and stored as .svs files via the Hamamatsu NanoZoomer NDP.serve 3 platform.

### Analysis of pTDP-43 pathology

All observations were made blind to clinical and demographic information. For each section, the presence and cellular localization of pTDP-43-immunoreactivity were recorded independently by 2 investigators (H.S., W.S.) blind to demographics and clinical information. Standardized staging criteria for LATE-NC pathology were applied as: stage 1, pTDP-43 pathology confined to the amygdala; stage 2, as stage 1 with addition of pathology in the hippocampal formation; or stage 3, in which pathology was as stage 2, with addition of pTDP-43 profiles in neurons of the middle frontal gyrus.^14–16,38,39^ Further, cellular localization of pTDP-43 was determined as neuronal cytoplasmic inclusion (NCI), glial cytoplasmic inclusion (GCI), dystrophic neurites (DN), and intranuclear inclusion (II), where its extent estimated as none, sparse (< 5 inclusions across the section), moderate (5 to 19 inclusions), and numerous (20 or more inclusions).

### Statistical analysis

Statistical analyses were performed using GraphPad Prism 10 (GraphPad Software Inc., La Jolla, CA), with between-group differences assessed using the 2-sample t-test or Fisher’s exact test, as appropriate. Statistical significance was set at 2-sided *P* < .05.

## Results

### pTDP-43 pathology is associated with older age and comorbid neurodegenerative disease

**Figure 1:**
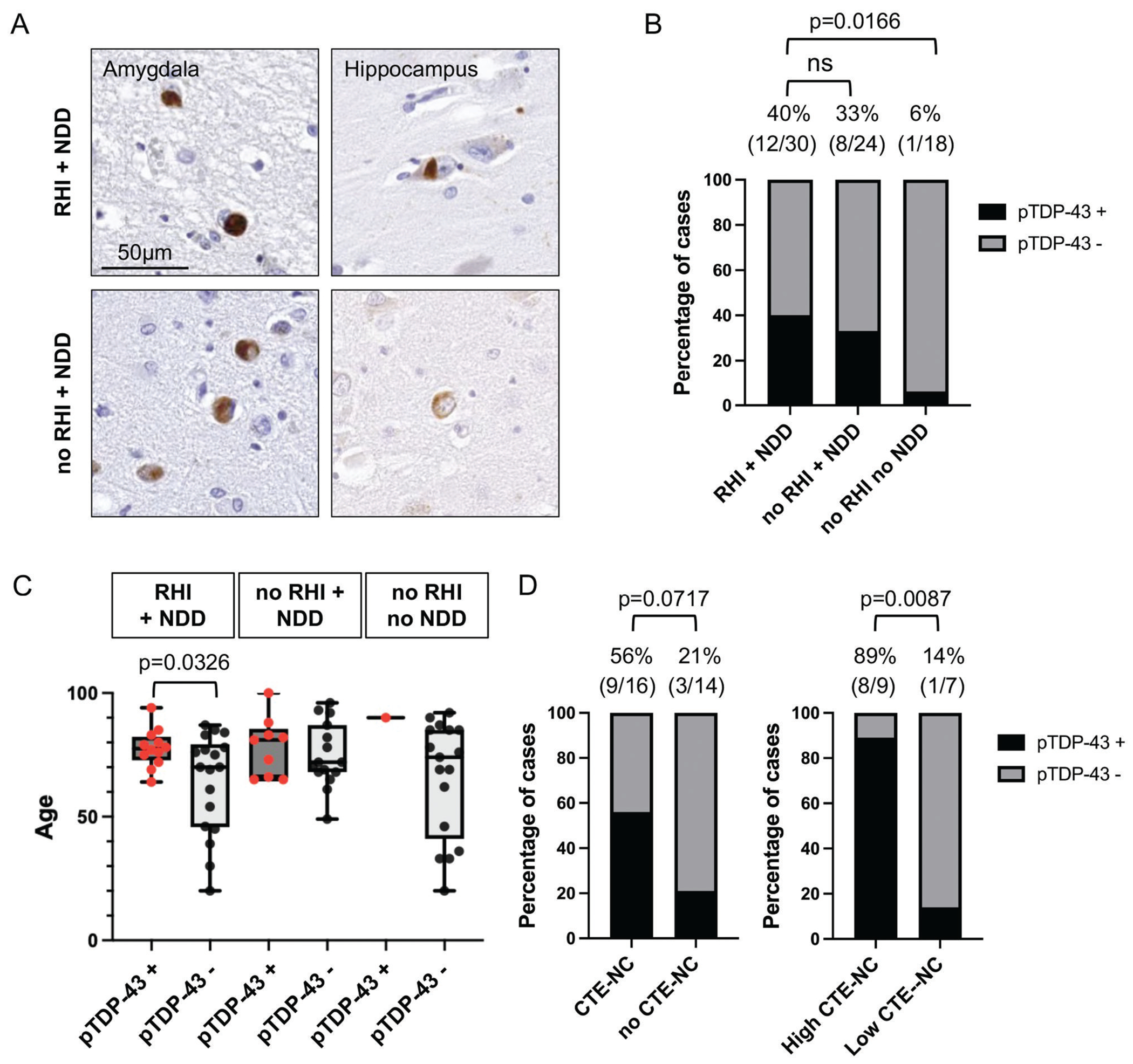
The presence of pTDP-43 pathology in relation to history of RHI exposure and CTE-NC. (A) Representative pTDP-43-immunoreactive neuronal cytoplasmic inclusions in amygdala and hippocampal regions (scale bar = 50 μm). (B) No difference in the prevalence of pTDP-43 pathology between RHI + NDD and no RHI + NDD group. In contrast, significantly more frequent pTDP-43 pathology in RHI + NDD compared with no RHI no NDD group. (C) Comparison in age distribution between pTDP-43 positive cases (red circles) and pTDP-43 negative cases. (D) No statistical difference in the prevalence of pTDP-43 pathology between CTE-NC-positive cases and -negative cases. Among those with CTE-NC, there was more frequent pTDP-43 pathology in individuals with high CTE-NC compared with low CTE-NC.

As described previously,^15,16^ TDP-43 proteinopathy was visualized in amygdala, hippocampal, and cortical sections as scattered, pTDP-43-immunoreactive neuronal cytoplasmic inclusions and neurites (Figure 1A). Only 1 out of 18 (6%) controls with no known RHI or NDD showed pTDP-43 pathology. In contrast, where co-existent NDD was present, pTDP-43 was more frequently encountered, although there was no notable difference in prevalence of pTDP-43 in cases with NDD with or without history of RHI (Figure 1B). Specifically, 12 of 30 (40%) RHI + NDD and 8 of 24 (33%) controls with no RHI + NDD showed immunoreactivity for pTDP-43 (*P* = .7778; Fisher’s exact test). Typically, pTDP-43 pathology was associated with older age across all cases and controls examined (mean age of 77.7 in cases with pTDP-43 pathology vs 63.8 without, *P* = .0326, RHI + NDD group; 74.8 vs 78.1, *P* = .5425, no RHI + NDD group), with no difference in age between RHI + NDD and controls with no RHI + NDD containing pTDP-43 pathology (*P* = .9186; 2 sample t-test) (Figure 1C). When present, the cellular localization of pTDP-43 inclusions was similar between groups and included NCI, GCI, and/or DN. Interestingly, among those with pTDP-43, individuals with RHI + NDD showed more pronounced pTDP-43 pathology, with all cases (12/12) exhibiting numerous inclusions, compared with those with no RHI + NDD, in whom only 3 of 8 showed numerous inclusions while the remainder had moderate to sparse pathology (*P* = .0036). There was no difference in the presence of pTDP-43 inclusions in the medulla in patients with RHI + NDD (5/12) vs those with no RHI + NDD (3/8).

Among RHI + NDD cases, a higher proportion of CTE-NC positive cases contained pTDP-43 pathology (9/16; 56%) than among CTE-NC negative cases (3/14; 21%), although this difference was not statistically significant (*P* = .0717) (Figure 1D). There was also no notable difference in the cellular localization and extent of pTDP-43 pathology between groups. Similarly, although the proportion of CTE-NC cases with pTDP-43 pathology was higher than among controls with no RHI + NDD, this was not significant (*P* = .1991). However, among CTE-NC cases, pTDP-43 pathology was more frequent among high (8/9) than among low stage CTE-NC cases (1/7; *P* = .0087; Figure 1D).

### High stage LATE-NC following RHI exposure is associated with CTE-NC

**Figure 2:**
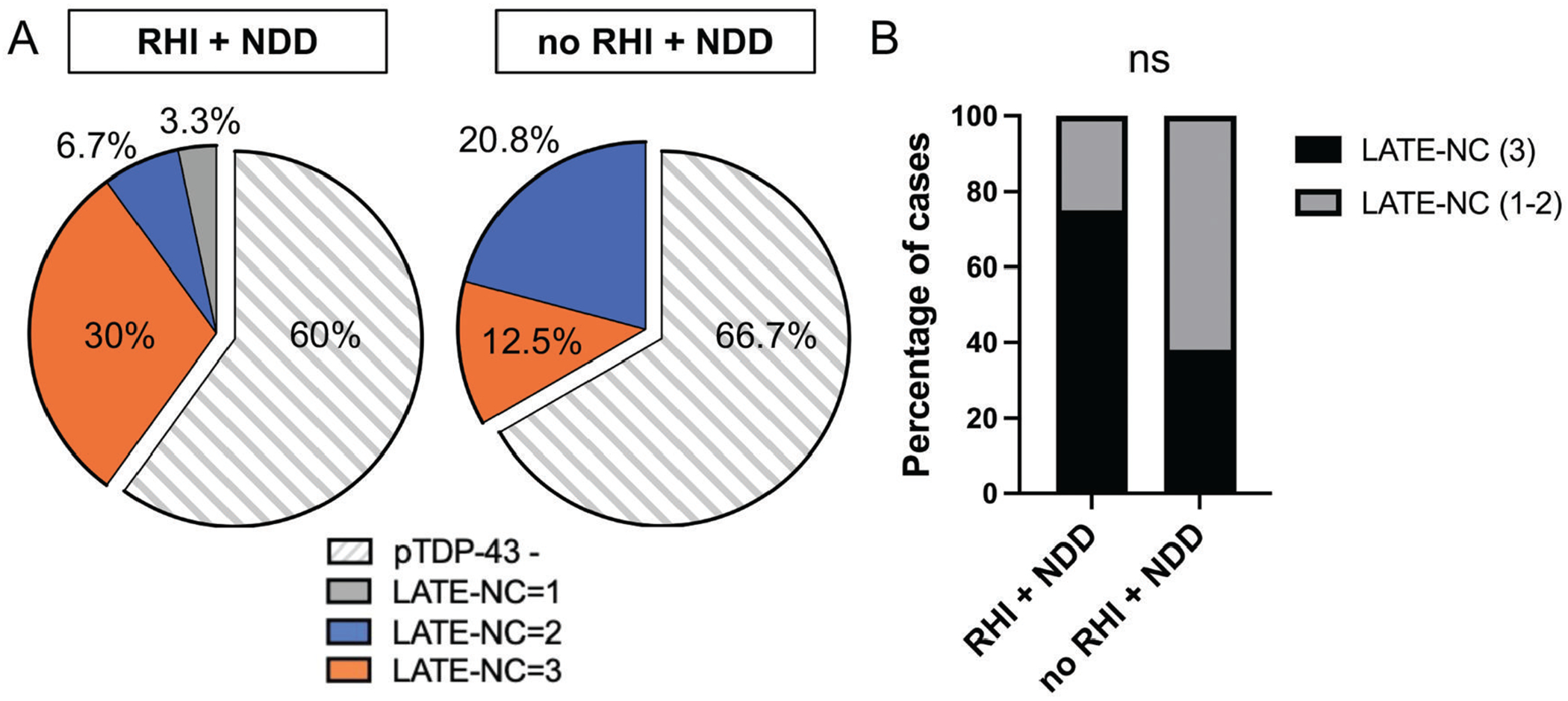
No association between history of RHI exposure and high LATE-NC stage 3. (A) Distributions of LATE-NC stages, including pTDP-43 negative cases, in RHI + NDD and no RHI + NDD groups. (B) No statistical difference in the percentage of LATE-NC stage 3 between RHI + NDD and no RHI + NDD group.

**Figure 3:**
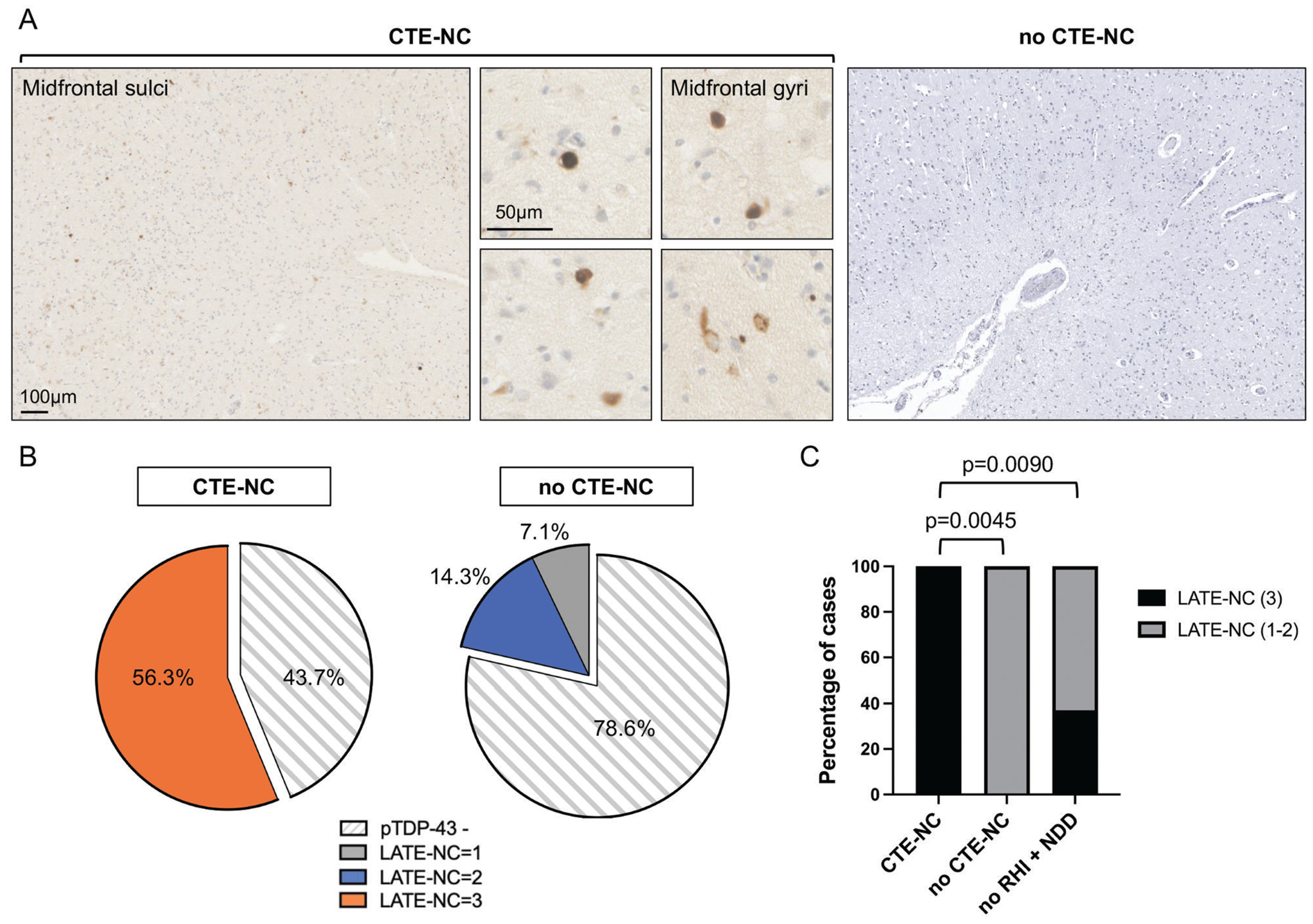
Higher prevalence of LATE-NC stage 3 in cases with co-existent CTE-NC. (A) Representative images of pTDP-43 pathology in the middle frontal gyrus in RHI + NDD cases with CTE-NC. In contrast, no observable pTDP-43 immunoreactivity in RHI + NDD cases with no CTE-NC. (B) Distributions of LATE-NC stages, including pTDP-43 negative cases, in CTE-NC positive and no CTE-NC groups. (C) Significantly greater prevalence of LATE-NC stage 3 in cases with CTE-NC vs cases with no CTE-NC. Significantly higher prevalence of LATE-NC stage 3 in those with CTE-NC than no RHI + NDD.

Assessing extent and distribution of pTDP-43 pathology by LATE-NC stage, dichotomized to stage 1 to 2 (limbic regions) or stage 3 (limbic plus middle frontal gyrus), revealed that where a case had other NDD pathologies, the proportion with LATE-NC stage 3 was greater in RHI (9/12; 75%) cases than in controls without RHI (3/8; 37.5%), although this was not significant (*P* = .1675). This may, in part, reflect limited statistical power given the modest sample size (Figure 2). However, among RHI + NDD cases, all those with LATE-NC stage 3 also showed co-existent CTE-NC (Figure 3), with prevalence of LATE-NC stage 3 higher in cases with CTE-NC than in age matched controls with no RHI + NDD (*P* = .0090). In addition, limbic pTDP-43 pathology was present in all CTE-NC cases with pTDP-43 inclusions in the middle frontal gyrus (9/9).

## Discussion

Leveraging unique, comprehensively assessed materials from individuals with known prior exposure to RHI and comparing to age matched controls with no known TBI/RHI exposure, either with or without NDD, we provide insights into the relationship between RHI and TDP-43 neuropathology. Specifically, while the overall prevalence of pTDP-43 pathology was similar among RHI patients and their age-matched controls with NDD, in those with RHI and documented CTE-NC, this pathology was more widely distributed. Thus, whereas pTDP-43 was typically localized to the amygdala and hippocampus in controls with NDD and in RHI patients without CTE-NC, this pathology was more often widespread and at a higher stage (LATE-NC stage 3) in patients with CTE-NC.

We recognize that the modest sample size from this study may limit its power to detect small effects. Nevertheless, our finding of a similar prevalence of pTDP-43 pathology among RHI patients, with or without CTE-NC, and their age-matched controls with NDD is consistent with other studies. Thus, as we describe here, a similar or lower prevalence of pTDP-43 pathology was reported in examinations of brains from retired professional soccer players and other contact sports participants.^6,8,27,28^ The study by Nicks et al further showed that the presence of pTDP-43 inclusions in the hippocampus was associated with hippocampal sclerosis in CTE-NC, extending previous examination of this relationship in aging and AD.^40^ Notably, our study elaborates on previous observations by providing a direct comparison with appropriate population controls, including patients with no history of RHI and those with or without co-existent NDD pathologies. Adopting this approach, we found that while the overall prevalence of pTDP-43 pathology among cases with RHI exposure was higher than that in age-matched controls without NDD, it was comparable to that observed in individuals with NDD but no history of RHI. In contrast, the burden of pTDP-43 pathology was greater in individuals with a history of RHI. There is also a notable trend toward a higher proportion of LATE-NC stage 3 in RHI cases than in those without RHI exposure, though this association needs to be confirmed in a larger cohort. Together with a previous examination of single TBI,^31^ these data support the notion that the presence of pTDP-43 pathology itself may not be associated with TBI or RHI exposure; this is consistent with findings from another community-based cohort showing no increased risk of pTDP-43 pathology in patients with TBI compared with those without TBI.^32^

Mixed proteinopathies are increasingly recognized in the context of TReND.^6,41^ Specifically, in CTE-NC, reports often describe co-existing neuropathological evidence of amyloid and *α*-synuclein pathologies, among others.^6,30,42,43^ Thus, it is conceivable that pTDP-43 in those with RHI history may simply reflect the co-existence of NDD. Intriguingly, although we observed a similar prevalence of pTDP-43 proteinopathy among those with RHI compared to NDD, where CTE-NC was present, this pathology was more frequent and typically more widespread, extending to neocortical regions, consistent with LATE-NC stage 3. This was particularly so with high stage CTE-NC and contrasted with age-matched controls with NDD, in which pTDP-43 pathology was more often limited to the amygdala and hippocampus. This observation is in line with reports documenting pTDP-43 pathology in wider NDD, such as in AD, in which pTDP-43 pathology is predominantly limbic (LATE-NC stages 1 or 2) and less often cortical (LATE-NC stage 3).^44^ Elsewhere, previous work suggests that among those with CTE-NC were selective cases with pTDP-43 inclusions affecting only the frontal cortex, but not limbic structures.^27^ Further pTDP-43 inclusions appeared distributed to the sulcal depth, mimicking the pattern of tau deposition seen in CTE-NC. These intriguing observations warrant examination in future work.

Damage to axons as diffuse axonal injury (DAI) is perhaps one of the most common pathologies following all severities of TBI, including RHI.^45–48^ Further, emerging clinical evidence from neuroimaging and fluid biomarker studies support DAI as a key contributor to both acute and chronic manifestations in patients with RHI.^49–53^ In the context of CTE-NC, axonal injury and degradation have been documented in widespread regions, including subcortical white matter.^30^ This is accompanied by tau hyperphosphorylation, which destabilizes axon microtubules, further contributing to axonal transport interruption. Interestingly, experimental evidence shows that sustained elevation of cytoplasmic TDP-43 could be associated with axonal transport interruption leading to compromised TDP-43 mRNA delivery to distal neuronal compartments.^54,55^ This suggests the possibility that axon disruption following TBI/RHI might contribute to the abnormal accumulation of pTDP-43.

In summary, this study reveals that while the prevalence of pTDP-43 pathology following RHI exposure might be consistent with that expected from NDD, in the context of CTE-NC, pTDP-43 is more often widely distributed, and consistent with highest stage LATE-NC. These observations add to our understanding of the complex neuropathologies of TReND and raise the intriguing possibility that this high stage pTDP-43 pathology of RHI might share a common underlying pathology with the unique tau pathologies seen in CTE-NC. Further studies to examine this relationship between CTE-NC and TDP-43 pathologies, particularly focusing on early stage CTE-NC, and the clinical associations with this pathology are required.

## Supplementary Material

Supplementary Material

Supplementary material is available at *Journal of Neuropathology & Experimental Neurology* online.

## Figures and Tables

**Table 1:** Case statistics.

Group	RHI + NDD	no RHI + NDD	no RHI no NDD
Number of cases	30	24	18
Median age (range)	76 years (20–94)	73 years (49–100)	75 years (20–92)
Males (%)	29 (96.7%)	11 (45.8%)	11 (61.1%)

Abbreviations: RHI, repetitive head impact; NDD, neurodegenerative disease.

**Table 2:** Case demographics.

Group	Case	Age	Sex	RHI exposure		CTE-NC stage
				Y/N	Type of sports or RHI history, if applicable	
RHI + NDD	1	20	M	Y	–	
	2	30	F	Y	Multiple concussions during cheer-leading and motor vehicle accidents	
	3	39	M	Y	–	
	4	45	M	Y	–	low
	5	46	M	Y	–	
	6	54	M	Y	American football/Wrestling	low
	7	61	M	Y	High school track and field, military combat training	
	8	64	M	Y	Soccer	high
	9	69	M	Y	Soccer	high
	10	69	M	Y	American football	low
	11	70	M	Y		
	12	70	M	Y	–	
	13	72	M	Y	American football/Wrestling	
	14	75	M	Y	American football	low
	15	75	M	Y	Soccer	high
	16	76	M	Y	Soccer	
	17	76	M	Y	–	
	18	77	M	Y	Soccer	
	19	77	M	Y	Soccer	
	20	78	M	Y	Soccer	high
	21	78	M	Y	Soccer	high
	22	79	M	Y	Soccer	high
	23	80	M	Y	Soccer/Rugby union/Boxing	low
	24	83	M	Y	Soccer	high
	25	83	M	Y	Soccer	low
	26	84	M	Y	–	low
	27	85	M	Y	Soccer	high
	28	85	M	Y	–	
	29	87	M	Y	American football	
	30	94	M	Y	Rugby union/Boxing	high
no RHI + NDD	31	49	M	N		
	32	61	M	N		
	33	65	M	N		
	34	65	M	N		
	35	65	F	N		
	36	66	M	N		
	37	68	M	N		
	38	68	F	N		
	39	69	F	N		
	40	70	F	N		
	41	72	F	N		
	42	72	M	N		
	43	73	F	N		
	44	78	M	N		
	45	81	M	N		
	46	82	M	N		
	47	82	F	N		
	48	83	F	N		
	49	87	M	N		
	50	88	F	N		
	51	92	F	N		
	52	93	F	N		
	53	96	F	N		
	54	100	F	N		
no RHI no NDD	55	20	M	N		
	56	33	M	N		
	57	33	F	N		
	58	36	F	N		
	59	46	M	N		
	60	62	M	N		
	61	69	M	N		
	62	69	M	N		
	63	74	F	N		
	64	76	F	N		
	65	78	M	N		
	66	85	M	N		
	67	85	M	N		
	68	85	F	N		
	69	87	M	N		
	70	90	M	N		
	71	90	F	N		
	72	92	F	N		

## Data Availability

The dataset supporting the conclusions of this article is included within the article.
