# Supplementary material for: Chronic traumatic encephalopathy neuropathologic change is associated with highest stage limbic-predominant age-related TDP-43 encephalopathy
Source: J Neuropathol Exp Neurol. Author manuscript; Available in PMC 2026 May 29. (PMC13220065; doi:10.1093/jnen/nlag040)
Supplement: Supplementary Material [file NIHMS2174301-supplement-Supplementary_Material.docx]

**Supplementary Table 1. Additional clinical data for all cases**

| **Group** | **Case** | **Clinical diagnosis (note)** | **Neuropathological diagnosis** |
| --- | --- | --- | --- |
| RHI + NDD | 1 | Major depressive disorder, post-concussion syndrome (death following MVA) | Acute diffuse axonal injury |
|  | 2 | Post-concussion syndrome, mood changes and headaches | Small parasagittal arachnoid cyst; sparse (pre)tangles in locus coeruleus and temporal lobe |
|  | 3 | N/A | Low ADNC |
|  | 4 | Acute psychosis | CTE-NC |
|  | 5 | Progressive memory problems, anxiety | Low ADNC |
|  | 6 | N/A | CTE-NC, ARTAG, PART |
|  | 7 | Vascular disease | Vascular brain injury, ARTAG, PART |
|  | 8 | AD | CTE-NC, intermediate ADNC, LATE-NC |
|  | 9 | N/A | CTE-NC, chronic cerebrovascular disease, low ADNC, TDP-43 proteinopathy |
|  | 10 | Hypertensive disease, sleep apnea, chronic lung disease, diabetes mellitus type II, arthritis | CTE-NC |
|  | 11 | Parkinson's disease dementia | DLB (neocortical), low ADNC, ARTAG |
|  | 12 | Dementia | Mixed pathology including high ADNC |
|  | 13 | AD | High ADNC, LATE-NC, ARTAG |
|  | 14 | Dementia | Remote contusions, CTE-NC, low ADNC, LATE-NC, ARTAG |
|  | 15 | Mixed AD and vascular dementia | CTE-NC, chronic cerebrovascular disease, high ADNC, ARTAG |
|  | 16 | AD | Intermediate ADNC, CAA, ARTAG, TDP-43 proteinopathy, moderate cerebrovascular disease, Lewy body pathology. |
|  | 17 | Dementia | Cortical contusions, high ADNC, ARTAG, CAA |
|  | 18 | AD | DLB (neocortical), high ADNC, TDP-43 proteinopathy, ARTAG, CAA, cerebrovascular disease (small vessel) |
|  | 19 | AD and vascular dementia | High ADNC, ARTAG, CAA, macro- and microvascular pathologies, limbic pattern Lewy body pathology |
|  | 20 | Vascular dementia or AD or mixed dementia | CTE-NC, high ADNC, LATE-NC, ARTAG, CAA, chronic cerebrovascular disease |
|  | 21 | N/A | CTE-NC, CAA, moderate cerebrovascular disease, intermediate ADNC, ARTAG |
|  | 22 | AD | CTE-NC, high ADNC, diffuse neocortical Lewy body pathology, LATE-NC, ARTAG, CAA, chronic cerebrovascular disease |
|  | 23 | Parkinson's disease dementia | CTE-NC, intermediate ADNC |
|  | 24 | Frontotemporal dementia and Parkinson's disease | CTE-NC, intermediate ADNC, ARTAG, TDP-43 proteinopathy, chronic cerebrovascular disease. |
|  | 25 | Normal pressure hydrocephalus | CTE-NC, ARTAG, chronic cerebrovascular disease |
|  | 26 | Dementia | CTE, ADNC, FTLD-TDP |
|  | 27 | AD and vascular dementia | ADNC, chronic cerebrovascular disease, CTE-NC, CAA, ARTAG |
|  | 28 | Vascular dementia | Low ADNC, white matter micro-infarcts |
|  | 29 | Mild cognitive changes | DLB (neocortical), PART, ARTAG |
|  | 30 | AD | CTE-NC, CAA, intermediate ADNC, ARTAG, LATE-NC |
| no RHI + NDD | 31 | ALS | ALS |
|  | 32 | AD | High ADNC, LATE-NC, olfactory LBD |
|  | 33 | Lewy body dementia | High ADNC, DLB (diffuse) |
|  | 34 | Corticobasal syndrome | CBD, low ADNC |
|  | 35 | AD | ADNC, Lewy body pathology, ARTAG |
|  | 36 | FTLD-bvFTD | Pick’s disease |
|  | 37 | Probable AD | High ADNC |
|  | 38 |  |  |
|  | 39 | Possible motor neuron disease with frontotemporal dementia; possible paraneoplastic syndrome | ADNC, CAA, chronic cerebrovascular disease |
|  | 40 | N/A | FTLD, ARTAG, AGD |
|  | 41 | FTLD-bvFTD | CBD, low ADNC |
|  | 42 | Lewy body dementia | DLB (neocortical), low ADNC, ARTAG |
|  | 43 | Probable AD | High ADNC, LATE-NC |
|  | 44 | N/A | ARTAG |
|  | 45 | AD | High ADNC, LATE-NC, LBD (amygdala), ARTAG |
|  | 46 | Parkinson's disease dementia | ADNC, Lewy body pathology, ARTAG, LATE-NC |
|  | 47 | AD | High ADNC |
|  | 48 | N/A | Lewy body pathology, vascular brain injury, ARTAG, PART, LATE-NC |
|  | 49 | Probable AD | High ADNC, cerebrovascular disease |
|  | 50 | N/A | Intermediate ADNC, LATE-NC |
|  | 51 | Probable AD | ADNC, Lewy body pathology, vascular brain injury |
|  | 52 | Probable AD | High ADNC, Lewy body pathology, ARTAG, metastatic carcinoma |
|  | 53 | N/A | Intermediate ADNC, vascular brain injury, ARTAG, moderate CAA |
|  | 54 | AD | High ADNC, LATE-NC, ARTAG |
| no RHI no NDD | 55 | N/A | N/A |
|  | 56 | N/A | N/A |
|  | 57 | N/A | N/A |
|  | 58 | N/A | N/A |
|  | 59 | N/A | N/A |
|  | 60 | N/A | N/A |
|  | 61 | N/A | N/A |
|  | 62 | N/A | N/A |
|  | 63 | N/A | N/A |
|  | 64 | N/A | N/A |
|  | 65 | N/A | N/A |
|  | 66 | N/A | N/A |
|  | 67 | N/A | N/A |
|  | 68 | N/A | N/A |
|  | 69 | N/A | N/A |
|  | 70 | N/A | N/A |
|  | 71 | N/A | N/A |
|  | 72 | N/A | N/A |

Abbreviations: AD, Alzheimer disease; ADNC, Alzheimer Disease Neuropathologic Changes; AGD, argyrophilic grain disease; ALS, amyotrophic lateral sclerosis; ARTAG, aging-related tau astrogliopathy; CAA, cerebral amyloid angiopathy; CBD, corticobasal degeneration; CTE-NC, chronic traumatic encephalopathy neuropathologic change; DLB, dementia with Lewy bodies; FTLD, frontotemporal lobar degeneration; MVA, motor vehicle accident; PART, primary age-related tauopathy
